# Exons 1–3 deletion in *FLCN* is associated with increased risk of pneumothorax in Chinese patients with Birt-Hogg-Dubé syndrome

**DOI:** 10.1186/s13023-023-02710-9

**Published:** 2023-05-12

**Authors:** Yue Wang, Mengru Cai, Xianliang Jiang, Guangyu Lv, Daiju Hu, Guofeng Zhang, Jinli Liu, Wei Wei, Jun Xiao, Bing Shen, Jay H. Ryu, Xiaowen Hu

**Affiliations:** 1 Department of Pulmonary and Critical Care Medicine, The First Affiliated Hospital of USTC, Division of Life Sciences and Medicine, University of Science and Technology of China, Hefei, 230001 Anhui China; 2 BengBu Medical College, Bengbu, Anhui China; 3 Jiangsu Key Laboratory of Molecular Medicine, Medical School, Nanjing University, Nanjing, 210093 Jiangsu China; 4 Department of Thoracic Surgery, The First Affiliated Hospital of USTC, Division of Life Sciences and Medicine, University of Science and Technology of China, Hefei, Anhui China; 5 Department of Dermatology, The First Affiliated Hospital of USTC, Division of Life Sciences and Medicine, University of Science and Technology of China, Hefei, Anhui China; 6 Department of Radiology, The First Affiliated Hospital of USTC, University of Science and Technology of China, Hefei, Anhui China; 7 Department of Urology, The First Affiliated Hospital of USTC, Division of Life Sciences and Medicine, University of Science and Technology of China, Hefei, Anhui China; 8 School of Basic Medicine, Anhui Medical University, Hefei, Anhui China; 9 Division of Pulmonary and Critical Care Medicine, Mayo Clinic, Rochester, MN USA

**Keywords:** Birt-Hogg-Dubé syndrome, Exons 1–3 deletion, Genotype–phenotype correlation, Pneumothorax

## Abstract

**Background:**

The pathogenic variants responsible for Birt-Hogg-Dubé syndrome (BHDS) in folliculin (*FLCN*) gene mostly consist of point mutations. Although large intragenic deletions/duplications have been reported in several case reports, the relationship between large intragenic deletions/duplications and phenotype in BHDS remains unclear.

**Methods:**

We retrospectively identified and reviewed patients with a large intragenic deletion spanning exons 1–3 and analyzed their phenotypic features to compare with those of point mutation carriers in our hospital from January 1, 2017 to August 31, 2022.

**Results:**

Twenty unique point mutations (including 4 novel mutations) were detected in 62 patients from 45 families (90%). Exons 1–3 deletion were identified in 8 patients from 5 families (10%) that resided in the same region, Feidong County of Anhui Province, China. Breakpoint analysis indicated that all the deletion breakpoints were flanked by Alu repeats. The prevalence of exons 1–3 deletion carriers in Feidong County was 8.1-times higher than that for BHDS in Anhui Province, suggesting a clustered phenomenon of exons 1–3 deletion. Significantly increased risk of pneumothorax was observed in those with exons 1–3 deletion compared with point mutations (91% vs. 58%,* p* value 0.047). The risk of renal cancer may be higher in those with exons 1–3 deletion than for those with point mutations (18% vs. 4%, *p* > 0.05).

**Conclusions:**

Large intragenic deletion of exons 1–3 in *FLCN* was identified as a local aggregation phenomenon in Feidong County, China, and was associated with a significantly higher risk of pneumothorax compared to those with point mutations.

**Supplementary Information:**

The online version contains supplementary material available at 10.1186/s13023-023-02710-9.

## Introduction

Birt-Hogg-Dubé syndrome (BHDS) is a rare autosomal dominant inherited disease caused by germline mutations in the *FLCN* gene [1]. It is characterized by varying expressivity of pulmonary, renal and cutaneous involvement. Multiple bilateral pulmonary cysts have been reported in over 80% of BHDS patients and predispose affected individuals to the occurrence of spontaneous pneumothorax [2, 3]. BHDS-associated renal tumors develop in 12–34% of cases and skin benign tumors occur in 68–84% of patients [4–7].

Mutations in the gene *FLCN,* located on chromosome 17p11.2, is associated with BHDS and consists of 14 exons [1]. To date, over 200 pathogenic variants have been reported worldwide, most of which are point mutations resulting in premature protein truncation [8, 9]. Toro et al. identified *FLCN* mutations in 88% of 58 clinically-diagnosed BHDS families by bidirectional DNA sequencing; large intragenic rearrangements may have been missed in the remaining 12% [4].

Kunogi et al. were the first to confirm the association of large intragenic deletions associated with BHDS in two affected individuals in 2010 [10]. Ding et al. identified three heterozygous large deletions in unrelated BHDS families, but genotype–phenotype correlations between large intragenic deletions versus point mutations were not comprehensively evaluated [11]. Further studies of large intragenic deletions in patients with BHDS seemed warranted.

We previously described the largest cohort of BHDS patients diagnosed in China, of which five patients from three unrelated families were identified to have a deletion encompassing exons 1–3 in *FLCN* [6]. In the current study, we aimed to report all the families with exons 1–3 deletion in *FLCN* evaluated at our hospital in an attempt to analyze their genotype–phenotype correlations compared with those carrying point mutations.

## Methods

### Study population

This single-centered, retrospective study was approved by the ethics committee of the First Affiliated Hospital of University of Science and Technology of China in Anhui Province (Number 2022-RE-436). We recruited patients diagnosed with BHDS in our Rare Lung Disease Clinic, supported by a multidisciplinary team, from January 1, 2017 to August 31, 2022 in this study. Informed consent was obtained from all patients but one died, which was waived. The study inclusion was a diagnosis of BHDS, which was based on the criteria proposed by the European BHDS consortium and required a patient to fulfill one major or two minor criteria [12]. The major criteria included: (1) At least five adult-onset fibrofolliculomas or trichodiscomas with at least one histologically confirmed; (2) Pathogenic *FLCN* germline mutation. The minor criteria: (1) Multiple bilateral, basally located lung cysts with no other apparent cause, with or without spontaneous pneumothorax; (2) Early onset (< 50 years), multifocal or bilateral renal cancer, or renal cancer of mixed chromophobe and oncocytic histology; (3) A first-degree relative with BHDS. Patients with the following conditions were excluded: age below 18 years, those with unclear diagnosis of BHDS or insufficient clinical data.

Clinical data of all patients including gender, age at diagnosis, smoking history, prior medical history (skin lesion, pneumothorax, and renal cancer), family history, imaging studies, genetic testing, and treatment were reviewed. Chest computed tomography (CT) results were assessed for the presence of pulmonary cysts and pneumothorax by a radiologist (Dr. WW) and two pulmonary physicians (Drs. YW, XH) independently. The total number (< 10, 10–20, > 20) and maximum diameter (< 2, 2–5, > 5 cm) of pulmonary cysts were recorded. The renal involvement was evaluated by ultrasound, abdominal CT, magnetic resonance imaging (MRI) or positron emission tomography-computed tomography (PET-CT). Skin manifestations were examined by an experienced dermatologist (Dr. JL) and skin biopsies were performed if the patient consented.

### Mutation analysis of the *FLCN* gene

Genomic DNA was extracted from peripheral blood leukocytes following standard protocols and performed by Sanger sequencing or next generation sequencing (NGS) strategy. The sequencing reactions were conducted as previously described [6, 13]. MLPA (Multiplex Ligation-Dependent Probe Amplification) assay was performed to detect for large intragenic deletions/duplications in *FLCN* and its up/downstream regions using the commercial kit P256-B1 *FLCN* (MRC-Holland, Amsterdam, Netherlands). The probes of the kit can target all 14 exons. MLPA reactions were performed by following the manufacturer’s instructions. The polymerase chain reaction (PCR) products were analyzed on an ABI 3130 Genetic Analyzer (Applied Biosystems). Data were analyzed using the Coffalyser software (MRC-Holland). The junction fragments adjacent to the deleted regions were amplified using the PCR Amplification Kit (Takara, China) with specially designed primers (F: CTGAGGGACACCAAGCACTC R: TGGGAAAGATGTTAATGGCCTA). PCR products were separated by 2% agarose gel electrophoresis. Bidirectional Sanger sequencing was performed. *FLCN* mutations were numbered based on Genbank accession numbers NM_144997.7 according to the HGVS nomenclature guideline (http://www.hgvs.org/mutnomen).

### Statistical analysis

Statistical analyses were conducted using SPSS version 25.0. Continuous variables were expressed as means and standard deviations and compared by independent sample t-test. Categorical variables were described as frequencies and percentages, and compared by the chi-square test and Fisher’s exact test. Binary logistic regression analysis was performed to identify risk factors for pneumothorax in BHDS. A value of *p* < 0.05 was considered statistically significant.

## Results

A total of 76 patients from 50 unrelated families were diagnosed as BHDS in our Rare Lung Disease Clinic from January 1, 2017 to August 31, 2022. All patients with BHDS were of Han ethnic group. Seventy patients were confirmed by genetic testing and the remaining six by clinical diagnosis. Among them, forty-seven were female and the ratio of male to female was 1:1.6. The average age of diagnosis was 44 ± 14 years (range, 18–76 years). Eight (11%) patients had a smoking history.

### Germline mutations of the *FLCN* gene

**Fig. 1:**
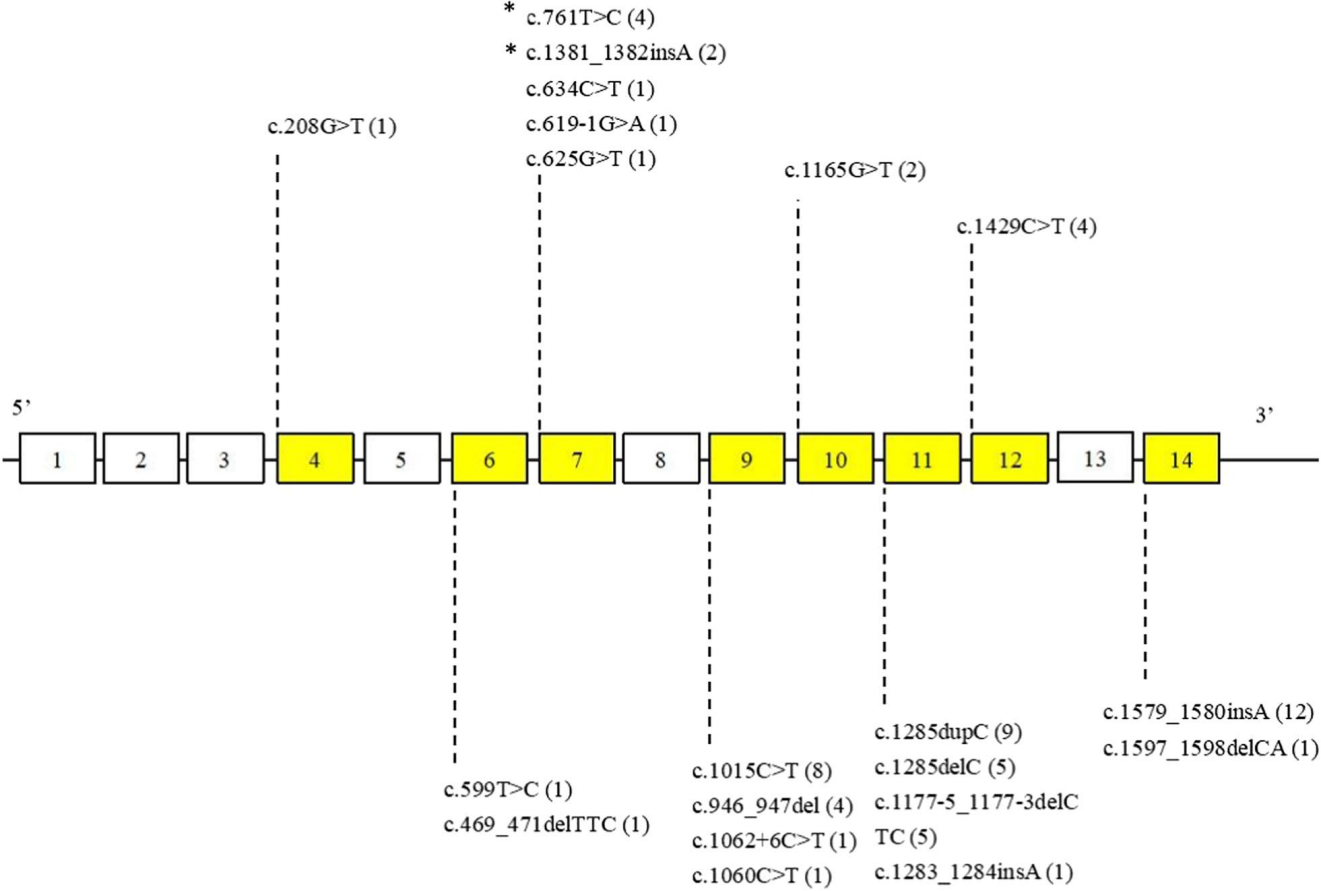
Point mutations at *FLCN* found in our cohort are listed. White boxes represent exons and yellow boxes represent exons with pathogenic point mutations in *FLCN*. The number of patients for each mutation is given in parentheses. *One patient in each of the three mutations did not undergo genetic testing, but the mutations were identified in their first-degree relatives

**Fig. 2:**
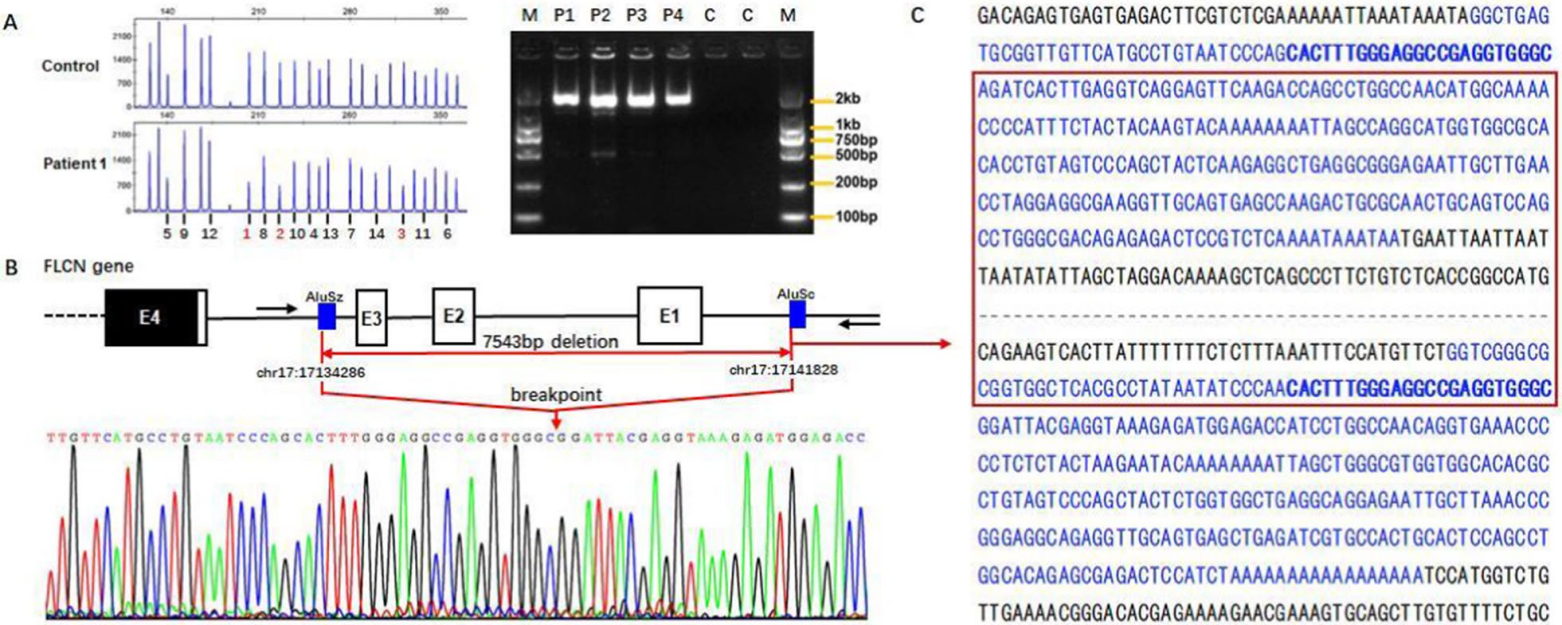
The results of identification of *FLCN* intragenic deletion in BHDS patients. **A** The electrophoretogram by MLPA on the left shows that the patient (F14-3) has a loss of heterozygosity in exon 1, 2 and 3 (in red); PCR products of junction fragments were separated by 2% agarose gel electrophoresis on the right. The characters and numbers above the lanes corresponded to the subjects (P1-P4) and controls (C1-C2). **B** and **C** The electrophoretogram and DNA sequence analysis of breakpoint junction show the 7,543 bp deletion mediated by the microhomology (blue bold bases in C) in Alu repeats (Alu SZ and Alu SC indicated by blue rectangular box in Fig. 2B and blue bases in C) span the exon 1 to 3 (E1-E3) of *FLCN* gene. The black arrows indicate the position of the primer for amplification of breakpoint junction in B

DNA samples from these 50 unrelated families were analyzed, of which unique pathogenic *FLCN* variants were identified in 62 patients from 45 families (90%) by Sanger sequencing (38 families) and NGS strategy (7 families), and three patients (12.2, 21.2 and 44.2) did not undergo genetic testing since their first-degree relatives carried germline *FLCN* mutations (Additional file 1: Table S1). The mutation spectrum revealed 20 different mutations, including seven nonsense, seven frameshift, three splice site, two missense, and one in-frame mutations (Fig. 1). A known mutational “hot spot”, c.1285dupC in exon 11, was detected in 9 patients from 7 families (14%) (Additional file 2: Fig. S1B). In addition, four heterozygous novel mutations were identified: c.761 T > C detected in three families, c.599 T > C in one family, c.1381_1382insA in one and c.1283_1284insA in one, respectively. The remaining 5 *FLCN* sequence-mutation negative families (10%) were detected to have a large intragenic deletion encompassing exons 1–3, of which 8 patients from 5 families were diagnosed by MLPA analysis (F13、F14、F15、F34、F40) (Fig. 2A). In addition, 3 patients (34.2, 34.3 and 34.4) in F34 were clinically diagnosed since they exhibited at least one of BHDS-related manifestations and the proband (34.1) was an exons 1–3 deletion carrier. The 1.8 kb PCR products of the junction fragment was separated by agarose gel electrophoresis, whereas the 9.3 kb wildtype sequence was too long to be amplified (Fig. 2A). Analysis of the sequencing results demonstrated one breakpoint to be located near chr 17: 17,134,286 and another breakpoint near chr 17: 17,141,828 with the estimated deletion size of 7,543 bp in length. Further analysis illustrated the involvement of AluSz and AluSc located on the up and downstream breakpoint junctions, respectively (Fig. 2B and C). A 23 bp stretch of microhomology in both Alu repeats suggested microhomology-mediated genomic recombination as a possible mechanism underlying in the deletion.

### The local aggregation phenomenon of exons 1–3 deletion

Interestingly, all 5 families harboring exons 1–3 deletion came from the same region, Feidong County, located near the center of Anhui Province; no kinship was identified among these families. Since nearly all BHDS patients in Anhui Province (including Feidong County) are evaluated and managed at our hospital, based on the most recent demographic data for Anhui Province at the end of 2021, the prevalence of BHDS in Anhui Province (population 61.13 million [14]) was estimated to be 1.24 cases per million. In Feidong County (population 1.09 million [15]), the carrier frequency of exons 1–3 deletion was 10.09 cases per million, which is 8.1 times higher prevalence of BHDS compared with that in Anhui population overall. The markedly increased frequency of exons 1–3 deletion carriers in a local region shows a clustered phenomenon of this variant in the *FLCN* gene.

### Genotype–phenotype correlations

Of 76 affected individuals, 11 cases were carriers of exons 1–3 deletion, while the other 65 harbored point mutations. The demographic characteristics of these subjects are summarized in Table 1. The average age at diagnosis for BHDS in intragenic deletion carriers was 47 ± 12 years (range, 19–73 years); 8 patients (73%) were females and only 1 had a history of smoking. There was no significant difference between the patients with exons 1–3 deletion and those with point mutations in age at diagnosis, gender distribution, and smoking history.

**Table 1:** Clinical characteristics of exons 1–3 deletion carriers and point mutations carriers

Characteristics	Exons 1–3 deletion (*N* = 11)	Point mutations (*N* = 65)	*P* value
Male/female	3/8	26/39	0.739
Age at diagnosis (yr)*	47 ± 12 (19–73)	44 ± 14 (18–76)	0.202
Smoking history (%)	1 (9)	7 (11)	1
Pulmonary manifestations (%)			
Cysts on chest CT	11/11 (100)	60/62 (97)	1
Number of cysts			
< 10	1 (9)	7 (12)	1
10–20	1 (9)	15 (25)	0.436
> 20	9 (82)	38 (63)	0.312
Size of maximum cysts (%)			
< 2 cm	0 (0)	12 (20)	0.191
2–5 cm	7 (64)	31 (52)	0.527
> 5 cm	4 (36)	17 (28)	0.721
PTX (%)	10/11 (91)	38/65 (58)	0.047
Age at onset of PTX (yr)*	36 ± 11 (19–58)	37 ± 12 (18–62)	0.753
Number of PTX episodes*	2 ± 3 (1–11)	2 ± 2 (1–7)	0.752
Renal cancer (%)	2/11 (18)	2/55 (4)	0.126
Skin manifestations (%)	8/9 (89)	47/60 (78)	0.674

*PTX* pneumothorax

^*^Age at diagnosis, age at onset of PTX, and number of PTX episodes are presented as mean ± SD (range)

**Fig.3:**
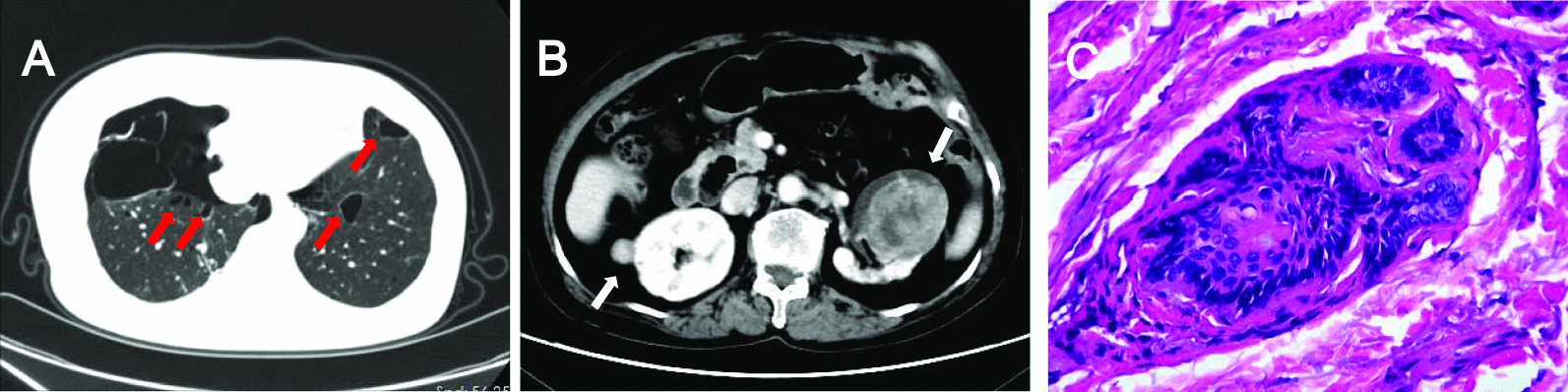
Pulmonary, kidney and skin involvements in BHDS patients with exons 1–3 deletion. **A** Chest CT showing multiple cysts distributed in the lower lungs (red arrows) and pneumothorax on the right. **B** Abdominal CT showing multiple bilateral kidney tumors with irregular edges (white arrows). **C** Hematoxylin and eosin staining of skin biopsy showing histologic features consistent with fibrofolliculoma (× 200)

All 11 patients with large deletion had multiple bilateral pulmonary cysts, predominantly distributed in the lower lung fields (Fig. 3A). The cyst morphology varied greatly, mainly of round, oval or irregular shapes. All patients had pulmonary cysts > 20 mm, while the largest cysts in four (36%) were > 50 mm with the maximum size at 74 mm. For total number of cysts, 9 patients (82%) had more than 20 pulmonary cysts, 1 (9%) had 10–20 cysts; the remaining patient (9%) with fewer than 10 cysts were a 19-year-old female who was treated by bullectomy after suffering an episode of pneumothorax.

Excluding 3 individuals without detailed CT data, multiple cysts were observed in 60 patients (97%) carrying point mutations including a 30-year-old male with only left-sided cysts. The largest cysts measured 20 to 50 mm in 31 patients (52%), and measured > 50 mm in 17 patients (28%). Sixty-three percent (38/60) of patients had more than 20 lung cysts and 25% (15/60) had 10–20 cysts. Although no significant difference in the sizes pulmonary cysts when comparing the two groups (exons 1–3 deletion versus point mutations), there was a tendency for the number of cysts in carriers of exons 1–3 deletion to be higher compared to those with point mutations (82% vs. 63%, *p* value 0.312).

Among 11 patients with intragenic deletion, 91% (10/11) experienced at least one episode of pneumothorax, with the mean age at the first episode of 36 ± 11 years (range, 19–58 years). The average number of pneumothorax was 2 ± 3 (range, 1–11). A female patient suffered the initial left pneumothorax at age 24 years and six episodes of ipsilateral recurrences, four episodes of contralateral pneumothoraces respectively in the following years despite three bullectomies and three chest tube drainage. A 43-year-old female has not developed pneumothorax yet, but her chest CT demonstrated more than 20 cysts with the largest cyst measuring 47 mm and located adjacent to the pleura.

58% (38/65) of sequence mutation-positive patients had experienced pneumothorax with an average number of 2 ± 2 episodes (range, 1–7). The mean age at onset of pneumothorax was 37 ± 12 years (range, 18–62 years). Mutation c.1285dupC was associated with a 33% risk (3/9) of pneumothorax. Significant difference was noted in pneumothorax risk between individuals with exons 1–3 deletion and those with point mutations (91% vs. 58%, *p* value 0.047). The risk of pneumothorax for subjects with exons 1–3 deletion was 2.8-times higher compared to those with c.1285dupC mutation (91% vs. 33%, *p* value 0.017). In addition, the relationship between history of pneumothorax and related factors mentioned above (smoking history, parameters related to pulmonary cysts, exons 1–3 deletion) was further examined by logistic regression analysis, which showed that only exons 1–3 deletion was associated with the presence of pneumothorax in BHDS (*p* value 0.028, odds ratio 10.000, 95% CI 1.280–78.117). However, there was no significant difference in the age at onset (*p* value 0.753) and the number of pneumothorax episodes between exons 1–3 deletion carriers and those with point mutation (*p* value 0.752).

Renal tumors were detected in 2 of 11 patients with exons 1–3 deletion (18%) (Fig. 3B). One patient underwent a partial nephrectomy for clear cell renal cell carcinoma at the age of 38 years. Due to poor performance status, another 73-year-old female was clinically diagnosed as having bilateral multifocal renal tumors by PET-CT. This patient underwent three renal artery embolization and died of advanced renal cancer 17 months later. Two patients with point mutations (4%) were found to have renal tumors as we previously described [6]. Large intragenic deletion carriers tended to be more likely to develop renal tumors compared to those with point mutations (18% vs. 4%, *p* value 0.126).

Eighty-nine percent (8/9) of carriers with exons 1–3 deletion manifested multiple pale or normal skin-colored papules on the face, neck, or upper trunk, of whom one underwent a biopsy and was confirmed to have fibrofolliculoma (Fig. 3C). Cutaneous involvement was seen in 78% (47/60) of point mutations carriers. Fibrofolliculomas were confirmed in 3 patients and trichodiscoma in 1. The prevalence of skin involvement in patients with large deletion and point mutations appeared to be similar (*p* value 0.674).

## Discussion

In current study, we identified by MLPA and breakpoint assay a unique large intragenic deletion encompassing exons 1–3 in 11 individuals with BHDS from 5 unrelated families; clinical features were also analyzed comprehensively by comparing to those with point mutations. Deletion of the promoter region (contained in exon 1) would significantly reduce *FLCN* expression from the mutant allele, which was consequently proved to be a pathogenic variant. Benhammou et al. defined the promoter region as a “hot spot” for *FLCN* deletions [16]. *FLCN* intragenic deletions/duplications reported around the world were summarized and compared with point mutations, but no genotype–phenotype correlation was found [17]. To our knowledge, our study is the first to evaluate the relationship between a large intragenic deletion and a particular phenotype in the Chinese population.

Although the prevalence of BHDS in the general population was recently calculated to be 1.86 cases per million, the exons 1–3 deletion carrier frequency in Feidong County was 10.1 per million, 5.4-fold higher, and 8.1-times higher than the prevalence of pathogenic *FLCN* variant carriers in Anhui Province [18]. Thus far, only 2 BHDS families with deletion spanning exons 1–3 have been reported, in Anhui Province and an adjacent province, Jiangsu, respectively; the two families shared a 3.0 Mb common haplotype [11].

Lagerstedt-Robinson et al. identified a splice site variant c.779 + 1G > T in 57% of BHDS families in the Swedish population, which showed a strikingly high carrier frequency in Swedish origin and was verified to be a founder mutation [19]. A previous study also revealed a similar phenomenon of mutation c.1062 + 2 T > G in the Danish population and defined it as a founder mutation [20]. In combination with previous studies, our data raise the suspicion that a founder effect might have contributed to the dramatically high prevalence of exons 1–3 deletion in localized region, i.e., Feidong County. Although further research is required to prove this hypothesis, we speculate that the five families and previously reported two families with exons 1–3 deletion are distant relatives. The local aggregation phenomenon of exons 1–3 deletion is predicted to emphasize that BHDS is more common than we have previously thought and that a founder effect might exist in populations with less genetic diversity.

BHDS is characterized by a wide phenotypic variability, leading to difficulty in timely recognition and diagnosis. There have been several studies in recent years examining the genotype–phenotype correlation in BHDS [21, 22]. In the current study, we analyzed the cases carrying exons 1–3 deletion and point mutations diagnosed in our hospital and discovered a novel genotype–phenotype correlation. The deletion spanning exons 1–3 was associated with a 91% risk for pneumothorax, 1.6-times higher than for those with point mutations (58%) and 2.8-times higher than for c.1285dupC (33%). Moreover, there seemed a higher risk of developing renal tumors in exons 1–3 deletion (18%) compared with those with point mutations (4%).

Multiple pulmonary cysts are usually the earliest and the most common manifestation in BHDS. Imaging features of varying sizes, irregular shape, and basal predominant distribution of pulmonary cysts are considered important clues to distinguish BHDS from other diffuse cystic lung diseases [23]. The mechanisms leading to the development pulmonary cysts are still incompletely understood. Several hypotheses have been proposed, including alterations in matrix metalloproteinases resulting from disturbed extracellular matrix homeostasis, and stretch theory based on defects in cell–cell adhesion [24, 25]. Previous studies illustrated that the removal of the putative *FLCN* promoter region resulted in reduced mRNA and protein expression levels of *FLCN*, which was confirmed to be pathogenic in pulmonary cyst formation [16, 26]. In this study, significant difference was not found for the size of pulmonary cysts, but the intragenic deletion tended to have more pulmonary cysts compared to those with point mutations.

Pulmonary cysts in BHDS are associated with a 50-times higher risk of spontaneous pneumothorax compared to the general population [27, 28]. An American survey from patients with BHDS showed that more than 70% of affected individuals experienced at least one episode of pneumothorax, consistent with the reports on the Japanese population [29, 30]. Pneumothorax was the main reason leading to the BHDS diagnosis in 42% of patients at our medical center [6]. A German study illustrated that the risk of pneumothorax was 1.6-fold higher in those with c.1300G > C variant and 2.1-fold higher in those with c.250-2A > G variant compared with 37% risk with c.1285dupC [31]. The present study revealed that more than 90% of patients with exons 1–3 deletion experienced pneumothorax with the youngest affected patient being just 19-years-old at the initial onset. This risk was significantly higher than 58% risk for pneumothorax in those with the point mutations and 33% risk in the subgroup with c.1285dupC variant.

Renal cancer is the most severe complication of BHDS, with a seven-fold increased risk compared to the general population [28]. Sattler et al. examined the relationship between the risk of renal cancer and the intragenic position of the mutation, but did not find a significant correlation [22]. The prevalence of renal cancer in our cohort was relatively low (5%). Exons 1–3 deletion was associated with 4.5-fold higher risk compared to those with point mutations, but the difference was not significant; limited sample size and lack of long-term follow-up might be partly responsible. There may also have been a referral bias since most BHDS patients were identified in the respiratory department. Future studies with larger cohorts recruited from multidisciplinary approach are needed to explore the relationship between renal cancer and specific *FLCN* mutations.

Skin involvement is regarded as a common feature of BHDS in both Asian and Caucasian populations, presenting as multiple, dome-shaped, and asymptomatic papules during the third to fourth decades [6, 32, 33]. A skin biopsy can assist physicians to establish a diagnosis together with pulmonary or renal manifestations [12]. We observed no correlation between skin lesions and the variant of exons 1–3 deletion in this study but skin biopsies were performed in only a minority of cases (lack of patient consent).

There are several limitations to this study. Firstly, as a single-centered study, the sample size is relatively small, which may lead to potential bias in statistical results; multi-centered studies with larger cohorts are required to clarify the genotype–phenotype relationship in the future. Due to the retrospective nature of this study, thorough family screening was lacking for some patients which prevented us from verifying the founder effect of exons 1–3 deletion further. In addition, since the study is based on cases identified mainly through a lung disease clinic although with a multidisciplinary team, a selection bias may have led to an underestimation of renal cancers.

## Conclusion

In conclusion, we identified a local aggregation phenomenon of large intragenic deletion spanning exons 1–3 in a region in the center of Anhui Province, China, which may represent a founder effect of *FLCN* mutation. Patients with exons 1–3 deletion were more likely to experience pneumothorax than point mutations carriers. Multi-centered prospective studies are needed to explore the relationship between renal cancer and large intragenic deletions.

## Supplementary Information

**Additional file 1: Table S1.** *FLCN* germline mutations detected in 50 BHDS families.

**Additional file 2: Fig. S1.** Genetic test revealed different *FLCN* mutations in 3 unrelated families.

## Data Availability

The datasets generated and analyzed for this study are not publicly available due to participant privacy but are available from the corresponding author upon reasonable request.

## Abbreviations

BHDS: Birt-Hogg-Dubé syndrome
*FLCN*: Folliculin
CT: Computed tomography
MRI: Magnetic resonance imaging
PET-CT: Positron emission tomography-computed tomography
PCR: Polymerase chain reaction
NGS: Next generation sequencing
MLPA: Multiplex ligation-dependent probe amplification
