# Supplementary material for: Exons 1–3 deletion in FLCN is associated with increased risk of pneumothorax in Chinese patients with Birt-Hogg-Dubé syndrome
Source: Orphanet J Rare Dis. 2023 May 12;18:115. doi: 10.1186/s13023-023-02710-9 (PMC10176890; doi:10.1186/s13023-023-02710-9)
Supplement: Supplementary file 1 — Additional file 1: Table S1. FLCN germline mutations detected in 50 BHDS families. [file 13023_2023_2710_MOESM1_ESM.docx]

**Table S1** *FLCN* germline mutations detected in 50 BHDS families

| **NO.** | **Family** | **Patient** | **Nucleotide change** | **Exon** | **Amino acid change** | **Method of detection** |
| --- | --- | --- | --- | --- | --- | --- |
| 1 | 1 | 1.1 | c.1165G > T | 10 | p.Glu389Ter | Sanger sequencing |
| 2 | 2 | 2.1 | c.208G > T | 4 | p.Glu70* | Sanger sequencing |
| 3 | 3 | 3.1 | c.1015C > T | 9 | p.Gln339Ter | Sanger sequencing |
| 4 |  | 3.2 | c.1015C > T | 9 | p.Gln339Ter | Sanger sequencing |
| 5 | 4 | 4.1 | c.469_471delTTC | 6 | p.Phe157del | Sanger sequencing |
| 6 | 5 | 5.1 | c.1015C > T | 9 | p.Gln339Ter | Sanger sequencing |
| 7 |  | 5.2 | c.1015C > T | 9 | p.Gln339Ter | Sanger sequencing |
| 8 | 6 | 6.1 | c.1579_1580insA | 14 | p.Arg527Glnfs*75 | Sanger sequencing |
| 9 |  | 6.2 | c.1579_1580insA | 14 | p.Arg527Glnfs*75 | Sanger sequencing |
| 10 |  | 6.3 | c.1579_1580insA | 14 | p.Arg527Glnfs*75 | Sanger sequencing |
| 11 |  | 6.4 | c.1579_1580insA | 14 | p.Arg527Glnfs*75 | Sanger sequencing |
| 12 |  | 6.5 | c.1579_1580insA | 14 | p.Arg527Glnfs*75 | Sanger sequencing |
| 13 |  | 6.6 | c.1579_1580insA | 14 | p.Arg527Glnfs*75 | Sanger sequencing |
| 14 |  | 6.7 | c.1579_1580insA | 14 | p.Arg527Glnfs*75 | Sanger sequencing |
| 15 | 7 | 7.1 | c.1597_1598delCA | 14 | p.Gln533Glufs*68 | Sanger sequencing |
| 16 | 8 | 8.1 | c.1177-5_1177-3delCTC | 11 |  | Sanger sequencing |
| 17 |  | 8.2 | c.1177-5_1177-3delCTC | 11 |  | Sanger sequencing |
| 18 | 9 | 9.1 | c.1285dupC | 11 | p.His429Profs*27 | Sanger sequencing |
| 19 | 10 | 10.1 | c.1177-5_1177-3delCTC | 11 |  | Sanger sequencing |
| 20 | 11 | 11.1 | c.1285delC | 11 | p.His429fs | Sanger sequencing |
| 21 | 12 | 12.1 | c.761T > C^a^ | 7 | p.L254P | Sanger sequencing |
| 22 |  | 12.2^†^ | *(c.761T > C^a^)* | *7* | *p.L254P* |  |
| 23 | 13 | 13.1 | Exons 1-3del | 1-3 |  | MLPA |
| 24 | 14 | 14.1 | Exons 1-3del | 1-3 |  | MLPA |
| 25 |  | 14.2 | Exons 1-3del | 1-3 |  | MLPA |
| 26 |  | 14.3 | Exons 1-3del | 1-3 |  | MLPA |
| 27 |  | 14.4 | Exons 1-3del | 1-3 |  | MLPA |
| 28 | 15 | 15.1 | Exons 1-3del | 1-3 |  | MLPA |
| 29 | 16 | 16.1 | c.1165G > T | 10 | p.Glu389Ter | Sanger sequencing |
| 30 | 17 | 17.1 | c.1285dupC | 11 | p.His429Profs*27 | Sanger sequencing |
| 31 | 18 | 18.1 | c.761T > C^a^ | 7 | p.L254P | Sanger sequencing |
| 32 | 19 | 19.1 | c.1285dupC | 11 | p.His429Profs*27 | Sanger sequencing |
| 33 | 20 | 20.1 | c.1285delC | 11 | p.His429fs | Sanger sequencing |
| 34 |  | 20.2 | c.1285delC | 11 | p.His429fs | Sanger sequencing |
| 35 | 21 | 21.1 | c.1381_1382insA^a^ | 7 | p.Ser461Lysfs*28 | Sanger sequencing |
| 36 |  | 21.2^†^ | *(c.1381_1382insA^a^)* | *7* | *p.Ser461Lysfs*28* |  |
| 37 | 22 | 22.1 | c.1062+6C > T | 9 |  | Sanger sequencing |
| 38 | 23 | 23.1 | c.1015C > T | 9 | p.Gln339Ter | Sanger sequencing |
| 39 |  | 23.2 | c.1015C > T | 9 | p.Gln339Ter | Sanger sequencing |
| 40 | 24 | 24.1 | c.946_947del | 9 | p.Ser316fs | Sanger sequencing |
| 41 | 25 | 25.1 | c.1015C > T | 9 | p.Gln339Ter | Sanger sequencing |
| **NO.** | **Family** | **Patient** | **Nucleotide change** | **Exon** | **Amino acid change** | **Method of detection** |
| 42 | 26 | 26.1 | c.634C > T | 7 |  | Sanger sequencing |
| 43 | 27 | 27.1 | c.761T > C^a^ | 7 | p.L254P | Sanger sequencing |
| 44 | 28 | 28.1 | c.1285delC | 11 | p.His429fs | Sanger sequencing |
| 45 | 29 | 29.1 | c.1429C > T | 12 | p.Arg477* | Sanger sequencing |
| 46 |  | 29.2 | c.1429C > T | 12 | p.Arg477* | Sanger sequencing |
| 47 | 30 | 30.1 | c.1283_1284insA^a^ | 11 | p.Pro428Hisfs*20 | Sanger sequencing |
| 48 | 31 | 31.1 | c.1285dupC | 11 | p.His429Profs*27 | Sanger sequencing |
| 49 |  | 31.2 | c.1285dupC | 11 | p.His429Profs*27 | Sanger sequencing |
| 50 | 32 | 32.1 | c.1579_1580insA | 14 | p.Arg527Glnfs*75 | Sanger sequencing |
| 51 |  | 32.2 | c.1579_1580insA | 14 | p.Arg527Glnfs*75 | Sanger sequencing |
| 52 |  | 32.3 | c.1579_1580insA | 14 | p.Arg527Glnfs*75 | Sanger sequencing |
| 53 |  | 32.4 | c.1579_1580insA | 14 | p.Arg527Glnfs*75 | Sanger sequencing |
| 54 | 33 | 33.1 | c.1579-1580insA | 14 | p.Arg527Glnfs*75 | Sanger sequencing |
| 55 | 34 | 34.1 | Exons 1-3del | 1-3 |  | MLPA |
| 56 |  | 34.2^†^ | *(Exons 1-3del)* | *1-3* |  |  |
| 57 |  | 34.3^†^ | *(Exons 1-3del)* | *1-3* |  |  |
| 58 |  | 34.4^†^ | *(Exons 1-3del)* | *1-3* |  |  |
| 59 | 35 | 35.1 | c.1285delC | 11 | p.His429fs | Sanger sequencing |
| 60 | 36 | 36.1 | c.1060C > T | 9 | p.Gln354Ter | Sanger sequencing |
| 61 | 37 | 37.1 | c.619-1G > A | 7 |  | Sanger sequencing |
| 62 | 38 | 38.1 | c.1285dupC | 11 | p.His429Profs*27 | Sanger sequencing |
| 63 | 39 | 39.1 | c.599T > C^a^ | 6 | p.L200P | Sanger sequencing |
| 64 | 40 | 40.1 | Exons 1-3del | 1-3 |  | MLPA |
| 65 | 41 | 41.1 | c.946_947del | 9 | p.S316fs | NGS |
| 66 | 42 | 42.1 | c.1015C > T | 9 | p.Gln339Ter | NGS |
| 67 | 43 | 43.1 | c.1285dupC | 11 | p.His429Profs*27 | NGS |
| 68 |  | 43.2 | c.1285dupC | 11 | p.His429Profs*27 | NGS |
| 69 | 44 | 44.1 | c.946_947del | 9 | p.S316fs | NGS |
| 70 |  | 44.2^†^ | *(c.946_947del)* | *9* | *p.S316fs* |  |
| 71 | 45 | 45.1 | c.1285dupC | 11 | p.His429Profs*27 | NGS |
| 72 | 46 | 46.1 | c.1429C > T | 12 | p.Arg477* | NGS |
| 73 | 47 | 47.1 | c.1177-5_1177-3delCTC | 11 |  | Sanger sequencing |
| 74 | 48 | 48.1 | c.1177-5_1177-3delCTC | 11 |  | Sanger sequencing |
| 75 | 49 | 49.1 | c.625G > T | 7 |  | Sanger sequencing |
| 76 | 50 | 50.1 | c.1429C > T | 12 | p.Arg477* | NGS |

^a^Novel mutation;^†^*FLCN* genetic testing was not performed, but the mutation identified in his/her family member was described; MLPA; Multiplex Ligation-Dependent Probe Amplification; NGS: Next Generation Sequencing.
