# Supplementary material for: Exons 1–3 deletion in FLCN is associated with increased risk of pneumothorax in Chinese patients with Birt-Hogg-Dubé syndrome
Source: Orphanet J Rare Dis. 2023 May 12;18:115. doi: 10.1186/s13023-023-02710-9 (PMC10176890; doi:10.1186/s13023-023-02710-9)
Supplement: Supplementary file 2 — Additional file 2: Fig. S1. Genetic test revealed different FLCN mutations in 3 unrelated families. [file 13023_2023_2710_MOESM2_ESM.docx]

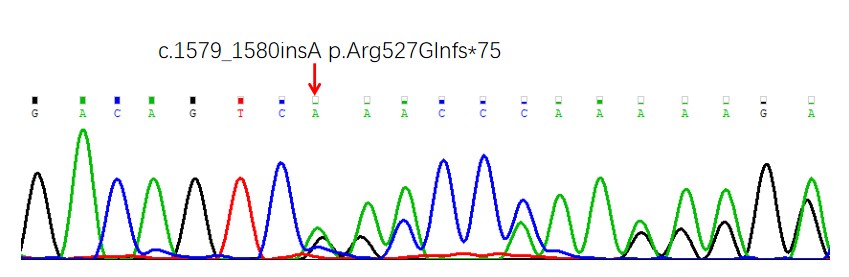

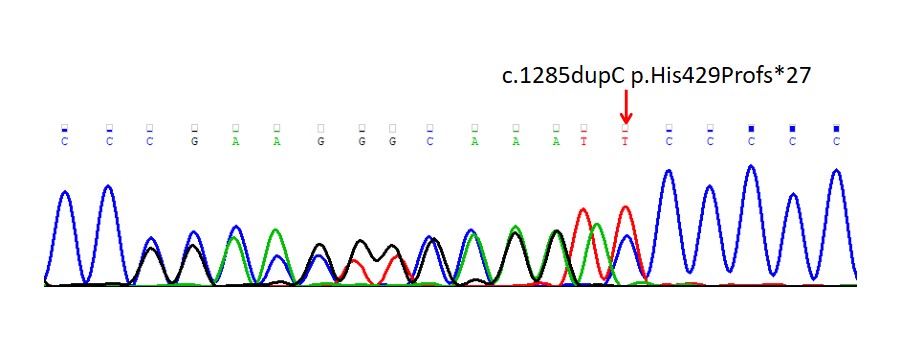

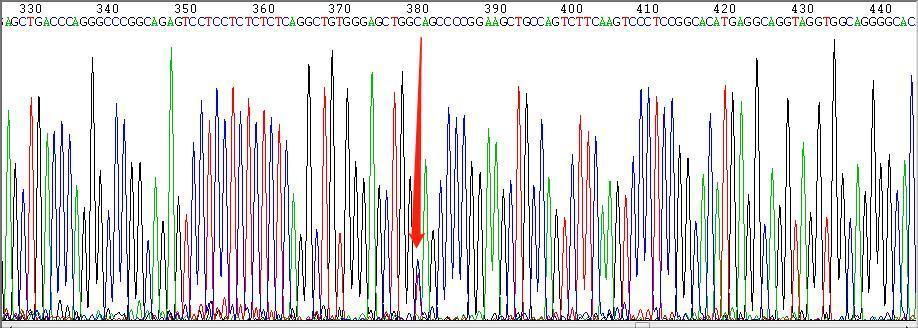


**A**

**B**

**C**

**Fig.S1** Genetic test revealed different *FLCN* mutations in 3 unrelated families. **A:** A frameshift mutation c.1579_1580insA was detected in F6 by Sanger sequencing. **B:** A frameshift mutation c.1285dupC was detected in F9 by Sanger sequencing. **C:** A nonsense mutation c.1015C > T was detected in F42 by next generation sequencing (exome sequencing).
